# Fluorescent Advanced Glycation End Products and Their Soluble Receptor: The Birth of New Plasmatic Biomarkers for Risk Stratification of Acute Coronary Syndrome

**DOI:** 10.1371/journal.pone.0074302

**Published:** 2013-09-13

**Authors:** Sergio Raposeiras-Roubín, Bruno K. Rodiño-Janeiro, Beatriz Paradela-Dobarro, Lilian Grigorian-Shamagian, José M. García-Acuña, Pablo Aguiar-Souto, Michel Jacquet-Hervet, María V. Reino-Maceiras, José R. González-Juanatey, Ezequiel Álvarez

**Affiliations:** 1 Servicio de Cardiología, Hospital Clínico Universitario de Santiago de Compostela, Santiago de Compostela, Spain; 2 Instituto de Investigación Sanitaria Santiago de Compostela, Santiago de Compostela, Spain; 3 Servicio de Cardiología, Hospital Meixoeiro, Vigo, Spain; 4 Departamento de Medicina, Universidad de Santiago de Compostela, Santiago de Compostela, Spain; University of Miami, United States of America

## Abstract

**Objective:**

Advanced glycation end products (AGEs) have pathophysiological implications in cardiovascular diseases. The aim of our study was to evaluate the prognostic value of fluorescent AGEs and its soluble receptor (sRAGE) in the context of acute coronary syndrome (ACS), both in-hospital phase and follow-up period.

**Methods:**

A prospective clinical study was performed in patients with debut’s ACS. The endpoints were the development of cardiac events (cardiac deaths, re-infarction and new-onset heart failure) during in-hospital phase and follow-up period (366 days, inter-quartile range: 273–519 days). 215 consecutive ACS patients admitted to the coronary care unit (62.7±13.0 years, 24.2% female) were included. 47.4% had a diagnosis of ST segment elevation myocardial infarction. AGEs and sRAGE were analysed by fluorescence spectroscopy and competitive ELISA, respectively. Risk scores (GRACE, TIMI, PURSUIT) were calculated retrospectively using prospective data. The complexity of coronary artery disease was evaluated by SYNTAX score.

**Results:**

The mean fluorescent AGEs and sRAGE levels were 57.7±45.1 AU and 1045.4±850.0 pg/mL, respectively. 19 patients presented cardiac events during in-hospital phase and 29 during the follow-up. In-hospital cardiac events were significantly associated with higher sRAGE levels (*p* = 0.001), but not long-term cardiac events (*p* = 0.365). Regarding fluorescent AGE the opposite happened. After multivariate analysis correcting by gender, left ventricular ejection fraction, glucose levels, haemoglobin, GRACE and SYNTAX scores, sRAGE was significantly associated with in-hospital prognosis, whereas fluorescent AGEs was significantly associated with long-term prognosis.

**Conclusions:**

We conclude that elevated values of sRAGE are associated with worse in-hospital prognosis, whereas high fluorescent AGE levels are associated with more follow-up events.

## Introduction

Reducing sugars can react non-enzymatically with the amino groups of protein to form Amadori products. These early glycation products undergo further complex reaction such as rearrangement, dehydration, and condensation to become irreversibly cross-linked, heterogeneous fluorescent derivatives, termed advanced glycation end products (AGEs) [1]. The binding of AGEs with their receptor (RAGE) results in diverse responses, including altered gene expression and cell migration and proliferation and activation of signalling pathways that are considered to play a pivotal role in the pathogenesis of atherosclerosis, heart failure, and other vascular complications [2]. Although the role of AGEs-RAGE interaction is deemed of great importance in diabetic vasculopathy, a growing body of evidence indicates that this signalling pathway can also play a role in non-diabetic atherosclerosis [3]. This hypothesis has been tested in rodent models of exaggerated neointimal expansion – a hallmark of coronary stenosis – which is triggered by chronic hyperglycaemia or oxidative stress; and provided the first clue that RAGE-dependent mechanisms of inflammatory and tissue perturbation were not limited to the diabetic state [4]. Blockade of RAGE significantly decreased vascular expression of adhesion molecules, pro-thrombotic species as tissue factor, and diminished antigen activity of matrix metalloproteinases [5], [6].

A soluble form of RAGE (sRAGE) can be measured in peripheral blood, which could result from the expression of a RAGE gene splice variant that encodes an amino-terminally truncated form of the receptor (esRAGE) and/or from the cleavage of the native membrane receptor (cRAGE) [7], [8]. The sRAGE levels have been found to be elevated in coronary artery diseases cases, as well as in patients with heart failure [9], [10].

In this study, we sought to gain a greater conceptual insight into the relationship between advanced glycation and coronary artery disease. In this regard, we explored the relationship between fluorescent AGEs and sRAGE with the presentation form and severity of acute coronary syndrome (ACS), the systolic function and the extension of coronary artery disease. Furthermore we investigated the predictive accuracy of fluorescent AGE and sRAGE for major adverse cardiac events in patients with acute myocardial infarction, both in-hospital and at the 1-year follow-up, and the relation with other biomarkers as well as classical risk scores.

## Materials and Methods

### Ethics Statement

Patients have been included in the study after written informed consent and the study and all the protocols used for the research were approved by the Ethical Committee for Clinical Investigations of Galicia (Spain) in accordance with the principles expressed in the 1975 Declaration of Helsinki.

### Study Population

**Figure 1:**
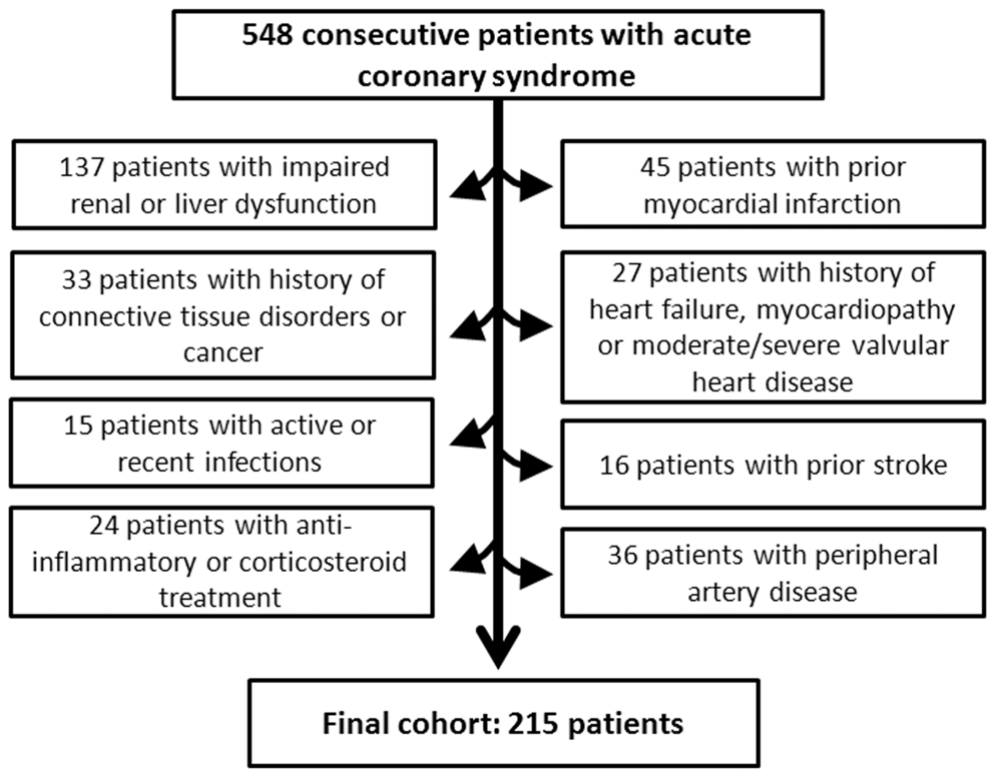
Consort diagram of the population included in the study.

Between October 2009 and January 2011, we performed an analysis of prospective data in consecutive patients admitted to our coronary care unit (CCU) with ACS and fitting our inclusion criteria. ACS was defined according to the European Society of Cardiology Guidelines [11]. Patients were classified as having acute myocardial infarction (AMI) with ST-segment elevation (STEMI) or ACS without ST-segment elevation (NSTE-ACS) [UA: unstable angina; NSTEMI: non-ST elevation AMI). The exclusion criteria of this study included pregnancy, previous myocardial infarction, a history of heart failure, myocardiopathy or moderate/severe valvular heart disease, prior stroke, arterial or venous thrombo-embolic disease, peripheral artery disease, impaired renal function (glomerular filtrate rate by MDRD-4<60 mL/min/1.73 m^2^), liver dysfunction, active or recent infections (last month), a history of inflammatory or connective tissue disorders, chronic or occasional (last 3 weeks) anti-inflammatory or corticosteroid treatment, cancer, haematological disorders, and previous major trauma or surgery (within 3 months). Thus, the final cohort was composed of 215 patients. Figure 1 represents a consort diagram of the population included in the study.

### Risk Scores

The GRACE [12] [Global Registry of Acute Coronary Events] (for in-hospital and for 6-months risk of death and myocardial infarction), TIMI [13], [14] [Thrombolysis in Myocardial Infarction] and PURSUIT [15] [Platelet glycoprotein IIb/IIIa in Unstable angina: Receptor Suppression Using Integrilin Therapy] risk scores were calculated from the initial clinical history and electrocardiogram, as well as from the values of laboratory parameters collected at admission, as a measurement of death risk. The complexity of coronary artery disease was evaluated by SYNTAX score [16]. All risk scores were calculated retrospectively (using prospective data) and separately by two persons who were blinded to the study results (agreement >98%).

### Measurement of Serum AGEs and sRAGE

AGEs were measured by quantitative fluorescence spectroscopy analysis of plasma according to the method of Munch et al [17]. By this method, we could measure some different AGE modifications at a time (crossline, fluorolink, pyrropyridine, vesperlysine, etc.), for which there are no immunological-based methods available nowadays. Plasma was aliquoted into black 96-well plates in duplicate and fluorescence (360/40∶460/40 nm; excitation:emission) was measured in a multi-mode microplate reader (Synergy 2, Biotek, Potton, United Kingdom) at room temperature to estimate the levels of fluorescent AGEs. Readings were subtracted from those of plasma-free wells to obtain measurements in arbitrary units (AU) and the mean of duplicated readings calculated. Plasma sRAGE levels were determined using a commercially available enzyme-linked immunosorbent assay kit (Quantikine; R&D systems, Minneapolis, MN, USA) according to the manufactureŕs protocol. Measurements were performed in duplicate and the results were averaged. This intra-assay and inter-assay coefficients of variation values were <5% and <8%, respectively.

### Follow-up and Endpoints

Events were defined as either cardiac death, as noted and confirmed by review of the death certificate and hospital chart or physician’s records, non-fatal myocardial infarction, as evidenced by the appropriate combination of symptoms, electrocardiogram, and enzyme changes, or admission due to heart failure. The strong study endpoint was the combination of cardiac death, reinfarction or heart failure. This endpoint was analysed both at the in-hospital phase and during the follow-up. The follow-up time was 366 days (inter-quartile range: 273–519 days). During the observation period, there was no dropout.

### Statistical Analysis

The statistical analyses were performed with SPSS (Statistical Package for the Social Sciences), version 17.0. The categorical or dichotomous variables were expressed as absolute values and percentages, and were compared with the Pearson 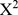 test. The continuous variables were described as the mean ± standard deviation (SD) when normally distributed or as the median and inter-quartile range for non-parametric data. Student *t* test was used for the comparisons of continuous variables between groups of patients. Continuous data from >2 groups were compared with ANOVA test. Receiver operating characteristic (ROC) curve analysis was performed to establish the diagnosis value of the different biomarkers to predict cardiac events. A binomial logistic regression model (with backward stepwise analysis) was used to evaluate the independent role of AGE and sRAGE as predictors of in-hospital adverse prognosis. A Cox proportional hazard analysis was carried out to assess the independent role of AGEs and sRAGE for predicting mortality during the follow-up. Adjusted odds, hazard ratios and 95% confidence intervals (CI) were presented. Kaplan Meier curves were performed to evaluate the prognostic value during follow-up of AGEs and sRAGE. Their results were analysed with log-rank test. A *p* value of <0.05 was considered statistically significant.

## Results

### Baseline Characteristics

The baseline characteristics of the final cohort, as well as the detailed characteristics of the events group, are shown in Table 1. The mean age was 62.7±13.0 years (24.2% female). 47.4% had a diagnosis of STEMI and 52.6% had NSTEMI (UA: 7.4%).

**Table 1:** Baseline characteristics of study population, stratified by presence of in-hospital and follow-up cardiac events.

Variables	Total population	In-hospital			Follow-up		
		Cardiacevents +	Cardiacevents −	*p*	Cardiacevents +	Cardiacevents −	*p*
***Demographic data***							
Age (years)	62.7±13.0	66.9±10.5	62.3±13.1	0.140	66.0±11.6	62.0±13.2	0.129
Female, %	24.2	47.4	21.9	**0.013**	13.8	26.0	0.156
***Medical history***							
Current smoking, %	34.9	26.3	35.7	0.523	34.5	35.4	0.459
Diabetes, %	25.6	36.8	24.5	0.239	34.5	23.8	0.217
Hypertension, %	46.0	47.4	45.9	0.904	62.1	44.8	0.083
Dyslipidaemia, %	44.2	57.9	42.9	0.208	48.3	43.1	0.602
***On admission data***							
NST-ACS, %	7.4	5.3	7.7	0.356	3.4	8.3	0.362
STEMI, %	47.4	63.2	45.9	0.151	55.2	47.0	0.411
Killip class ≥ II, %	13.5	68.4	8.2	**0.001**	31.0	9.9	**0.002**
Atrial fibrillation, %	7.4	5.3	7.7	0.705	13.8	6.5	0.177
***Laboratory data***							
Haemoglobin (g/dL)	14.3±1.6	13.3±1.9	14.5±1.5	**0.003**	13.6±1.7	14.5±1.5	**0.009**
Blood cell count (/mL)	9.7 (8.0–12.6)	9.7 (7.9–12.5)	11.7 (8.1–17.2)	0.078	9.8 (8.1–12.5)	9.4 (7.5–14.2)	0.893
LDL (mg/dL)	116.3±39.0	121.4±53.6	115.9±37.5	0.694	115.6±41.8	117.3±38.6	0.843
TPI peak (ng/mL)	13.9 (3.3–59.2)	12.9 (3.1–52.2)	49.1 (10.2–154.5)	**0.021**	12.3 (2.7–50.4)	57.9 (7.2–156.3)	**0.007**
***Risk Scores***							
TIMI	2.7±1.1	3.3±1.0	2.6±1.0	**0.011**	2.9±1.0	2.6±1.1	0.110
PURSUIT	9.6±4.1	11.6±3.6	9.4±4.2	**0.025**	11.5±3.2	9.3±4.2	**0.002**
GRACE	135.3±37.1	166.7±46.6	132.3±34.7	**0.001**	115.4±30.3	97.2±28.7	**0.002**
SYNTAX	14.2±7.3	18.6±5.6	13.8±7.4	**0.006**	16.1±8.4	13.7±7.2	0.118
***Glycations products***							
Glucose (mg/dL)	124.0 (104.0–170.0)	177.0 (139–0–347.0)	122.0 (103.2–158.0)	**0.001**	151.0 (113.5–260.5)	122.0 (103.5–157.5)	**0.025**
Fructosamine (mg/dL)	172.0 (148.0–209.2)	180.0 (139.0–335.5)	172.0 (148.0–208.5)	0.461	205.0 (159.0–311.0)	170.0 (148.0–197.0)	0.052
HbA1c, %	5.7 (5.4–6.2)	5.7 (5.5–8.4)	5.7 (5.4–6.1)	0.295	5.9 (5.5–8.0)	5.6 (5.4–6.0)	**0.015**
Fluorescent AGE (AU)	44.0 (34.0–55.5)	43.0 (38.0–51.0)	44.2 (33.6–56.0)	0.727	51.0 (40.2–157.7)	43.0 (32.7–53.2)	**0.002**
sRAGE (pg/mL)	833.0 (539.3–1256.5)	1709.0 (1130.0–2398.0)	809.3 (522.2–1126.0)	**0.001**	985.0 (533.7–1702.0)	827.0 (523.7–1251.0)	0.388
***Procedural characteristics***							
LVEF ≤45%, %	18.1	63.2	13.8	0.001	44.8	13.8	**0.001**
Multi-vessel disease, %	51.2	63.2	50.0	0.273	62.1	48.6	0.179
PCI, %	78.2	78.9	78.1	0.929	75.9	79.0	0.702
Complete revascularisation,%	80.0	78.9	89.5	0.276	78.3	80.3	0.823
***Discharge Treatments***							
Aspirin, %	100	–	–	–	100	100	–
Clopidogrel, %	99.0	–	–	–	100	98.9	0.564
B-blockers, %	74.3	–	–	–	72.4	74.6	0.804
ACEI/ARB II, %	77.7	–	–	–	86.2	76.3	0.431
Statins, %	94.3	–	–	–	95.5	94.2	0.809

**ACEI/ARB II:** Angiotensin-converting Enzyme Inhibitors/Angiotensin II receptor blockers. **BMI**: Body Mass Index. **LVEF**: Left Ventricular Ejection Fraction. **PCI**: Percutaneous Coronary Intervention. **STEMI**: ST-elevation myocardial infarction. **UA**: Unstable Angina.

The mean circulating fluorescent AGEs and sRAGE levels were 44.0 (34.0–55.5) AU and 833.0 (539.3–1256.5) pg/mL, respectively. There was no significant relation between these two variables (*p* = 0.705) and none of them were associated with diabetes mellitus.

### Glycation Products and ACS

**Figure 2:**
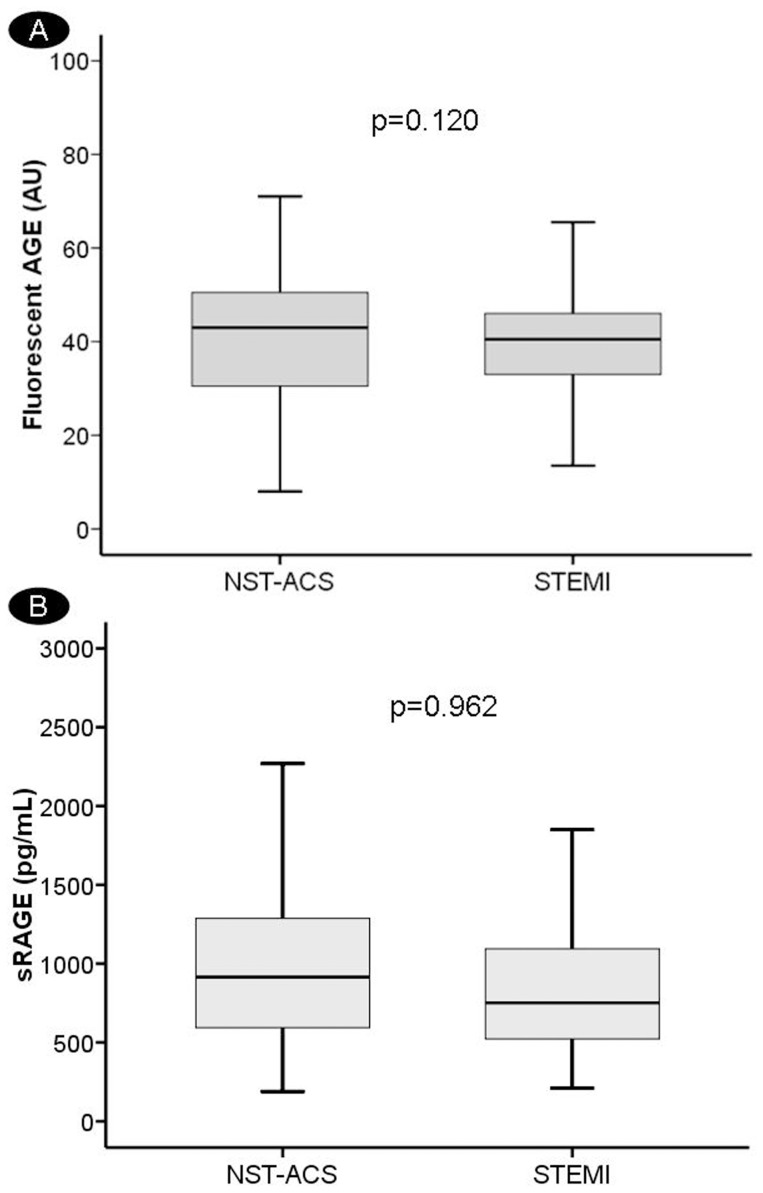
Relation of fluorescent AGEs and sRAGE with the type of ACS. Box plots for fluorescent AGE (A) and sRAGE (B) plasma levels for non ST-segment elevation acute coronary syndrome patients (NST-ACS) and ST-segment elevation myocardial infarction (STEMI).

There were no significant differences in fluorescent AGEs and sRAGE plasma levels among the different ACS subtypes (Figure 2) (AGE = 48.7 (41.7–52.6), 45.5 (32.0–62.2) and 42.5 (33.7–51.0) AU *vs*. sRAGE = 836.7 (438.0–1308.5), 988.0 (602.2–1290.0) and 777.0 (517.3–1118.0) pg/mL, *p*>0.05, for UA, NSTEMI and STEMI, respectively), although the percentage of NST-ACS patients (NSTEMI and UA) were higher in the higher quartiles of fluorescent AGEs (Table 2).

**Table 2:** Baseline characteristics based on quartiles of fluorescent AGE.

	Q1	Q2	Q3	Q4	
	≤34.0 AU(n = 53)	34.1–44.0 AU(n = 54)	44.1–55.5 AU(n = 54)	>55.5 AU(n = 54)	*p value*
***Demographic data***					
Age (years)	63.7±11.3	62.4±11.8	61.9±13.3	62.8±15.3	0.911
Female, %	22.6	29.6	21.8	22.6	0.758
***Medical history***					
Current smoking, %	22.6	37.0	43.6	35.8	0.097
Diabetes, %	20.8	27.8	25.5	28.3	0.803
Hypertension, %	47.2	31.5	54.5	50.9	0.080
Dyslipidaemia, %	39.6	38.9	49.1	49.1	0.550
***On admission data***					
STEMI, %	47.2	61.1	47.3	34.0	**0.048**
Killip class ≥ II, %	15.1	16.7	12.7	9.4	0.715
Atrial fibrillation, %	9.4	7.4	3.6	9.4	0.622
***Laboratory***					
Haemoglobin (g/dL)	14.4±1.4	14.4±1.4	14.5±1.5	14.2±2.0	0.686
Blood cell count (/mL)	10.0 (7.6–12.1)	10.3 (8.6–13.5)	9.9 (8.1–12.4)	9.4 (7.8–12.4)	0.844
LDL (mg/dL)*	112.8±35.9	122.1±42.6	116.1±34.3	114.2±42.7	0.666
TPI peak (ng/mL)*	21.4 (3.1–69.4)	18.9 (2.3–54.4)	11.8 (3.8–70.0)	10.8 (3.3–41.4)	0.595
***Procedural characteristics***					
LVEF <45%, %	20.8	24.1	17.0	18.1	0.320
Multi-vessel disease, %	43.4	42.6	60.0	64.2	**0.046**
Complete revascularisation, %	86.4	81.3	81.6	70.5	0.290

Abbreviations as in table 1.

Patients were divided according to the fluorescent AGEs quartiles at admission (Q1≤34.0 AU; Q2∶34.1–44.0 AU; Q3∶44.1–55.5 AU; and Q4>55.5 AU; Table 2). There was a slight trend to more smoking habits and more hypertension in the upper quartiles of fluorescent AGEs (*p = *0.097 and *p* = 0.080, respectively). There was no association between fluorescent AGEs and cholesterol. Fluorescent AGEs tended to correlate with fructosamine (r = 0.143, *p* = 0.072) and HbA1c (r = 0.129, *p* = 0.082), but not with glucose levels (r = 0.091, *p* = 0.186). High fluorescent AGEs levels were associated with the presence of multi-vessel disease and more vessels affected, but not with the worst Killip class or lower LVEF. Likewise, there was no association between fluorescent AGEs and infarct size (troponin I peak).

Patients were also divided on the basis of sRAGE quartiles at admission (Q1≤538.4 pg/mL; Q2∶538.5 to 826.9 pg/mL; Q3∶827.0–1255.0 pg/mL; and Q4>1255.0 pg/mL; Table 3). No differences among the distribution of classical risk factors were found between each quartil of sRAGE levels. Neither the lipid profile nor glycaemic values (glucose, fructosamine, HbA1c, and AGEs) were related to sRAGE levels. There was a significant increase in sRAGE levels in relation to the severity of Killip functional class at admission. The percentage of left ventricular dysfunction and atrial fibrillation was significantly higher with increasing sRAGE levels (*p* = 0.038 and *p* = 0.045, respectively), whereas the mean values of haemoglobin were lower. Infarct size, as measured by peak troponin I, was correlated with sRAGE levels (r = 0.306, *p*<0.001). Clinical outcomes (in-hospital and follow-up cardiac events) are displayed in Table 4.

**Table 3:** Baseline characteristics based on quartiles of sRAGE.

	Q1	Q2	Q3	Q4	
	≤538.4 pg/mL(*n* = 54)	538.5–826.9 pg/mL(*n* = 53)	827.0–1255.0 pg/mL(*n* = 54)	>1255.0 pg/mL(*n* = 54)	*p* value
***Demographic data***					
Age (years)*	60.7±12.9	61.9±12.3	62.3±14.7	65.8±11.6	0.201
Female, %	16.7	20.8	25.9	33.3	0.207
***Medical history***					
Current smoking, %	33.3	32.1	38.9	35.2	0.535
Diabetes, %	22.2	20.8	25.9	33.3	0.444
Hypertension, %	40.7	47.2	46.3	50.0	0.806
Dyslipidaemia, %	38.9	41.5	51.9	44.4	0.561
***On admission data***					
STEMI, %	53.7	58.5	38.9	38.9	0.085
Killip class ≥ II, %	9.3	11.3	7.4	25.9	**0.019**
Atrial fibrillation, %	0.0	5.7	13.0	11.1	**0.045**
***Laboratory***					
Haemoglobin (g/dL)	14.7±1.4	14.7±1.3	14.3±1.7	13.8±1.7	**0.004**
Blood cell count (/mL)	10.6 (8.2–13.3)	9.9 (8.3–13.7)	9.2 (7.3–11.4)	9.4 (8.1–12.5)	0.270
LDL (mg/dL)	123.8±41.1	119.1±39.1	106.7±31.9	115.8±42.3	0.173
TPI peak (ng/mL)	16.6 (5.3–41.2)	31.1 (4.2–105.1)	12.4 (2.0–46.0)	9.4 (3.6–108.6)	0.338
***Procedural characteristics***					
LVEF <45%, %	11.1	11.3	20.4	29.6	**0.038**
Multi-vessel disease, %	57.4	49.1	46.3	57.4	0.546
Complete revascularisation, %	77.3	79.6	84.1	79.2	0.875

Abbreviations as table 1.

**Table 4:** Clinical outcomes based on quartiles of sRAGE and fluorescent AGE.

PROGNOSTIC VALUE	sRAGE levels					Fluorescent AGE levels				
	Q1	Q2	Q3	Q4	*p*	Q1	Q2	Q3	Q4	*p*
**In-Hospital Events**	0.0	3.8	5.6	25.9	**0.001**	7.5	11.1	12.7	3.8	0.365
Cardiac death	0.0	5.7	1.9	1.9	0.263	3.8	1.9	1.8	1.9	0.885
Reinfarction	0.0	0.0	3.7	9.3	**0.020**	1.9	3.7	5.5	1.9	0.680
Heart Failure	0.0	0.0	0.0	18.5	**0.001**	3.8	5.6	9.1	0.0	0.156
**Follow-up events**	14.8	12.0	13.2	15.1	0.965	5.7	9.6	17.0	23.1	**0.048**
Cardiac death	1.9	2.0	3.8	0.0	0.568	1.9	0.0	1.9	3.8	0.560
Reinfarction	11.1	6.0	7.5	7.5	0.802	1.9	5.8	9.4	15.4	0.073
Heart Failure	5.6	4.0	7.5	7.5	0.895	3.8	3.8	5.7	9.6	0.537

No significant association was found between fluorescent AGEs and sRAGE levels and treatment at discharge (anti-platelets, B-blockers, ACEI/ARB-2 and statins).

### Glycation Products and Increasing Cardiovascular Risk

Clinical outcomes are shown in Table 4. Nineteen patients (8.8%) presented cardiac events during the in-hospital phase (5 cardiac deaths, 7 re-infarctions and 9 new-onset heart failure). During the follow-up, 29 patients (13.8%) presented cardiac events (4 cardiac death, 17 re-infarctions and 12 heart failure admissions).

**Figure 3:**
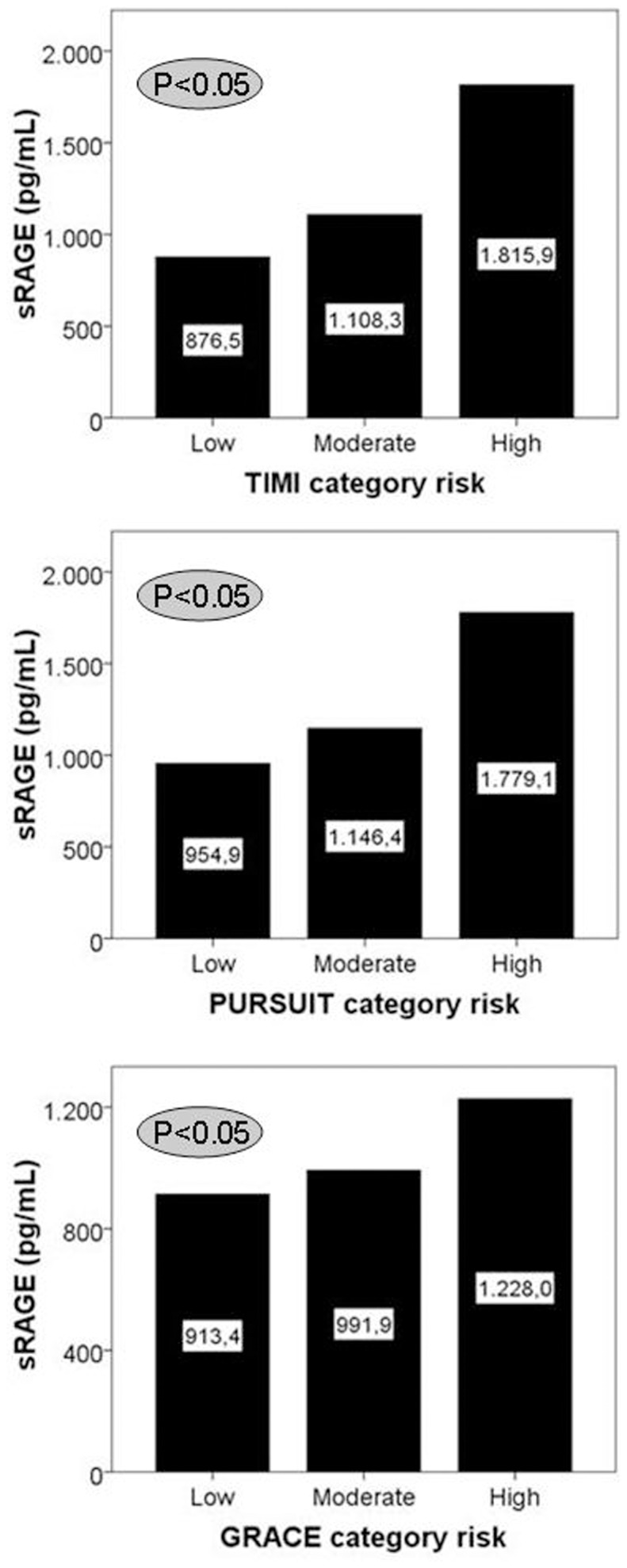
Relation of sRAGE levels and risk categories of different ACS scores. Columns represent mean values (indicated in numbers) for sRAGE plasma levels in each category of the indicated scores.

In-hospital cardiac events were significantly associated with higher sRAGE levels (*p* = 0.001), but not with long-term cardiac events (*p* = 0.365). This is consistent with significant correlations between sRAGE levels and the predicted cardiovascular in-hospital risk for each of the four risk scoring schemes as follows: TIMI (r = 0.210; *p* = 0.002), PURSUIT (r = 0.166; *p* = 0.015) and GRACE (r = 0.194; *p* = 0.004; Figure 3).

**Figure 4:**
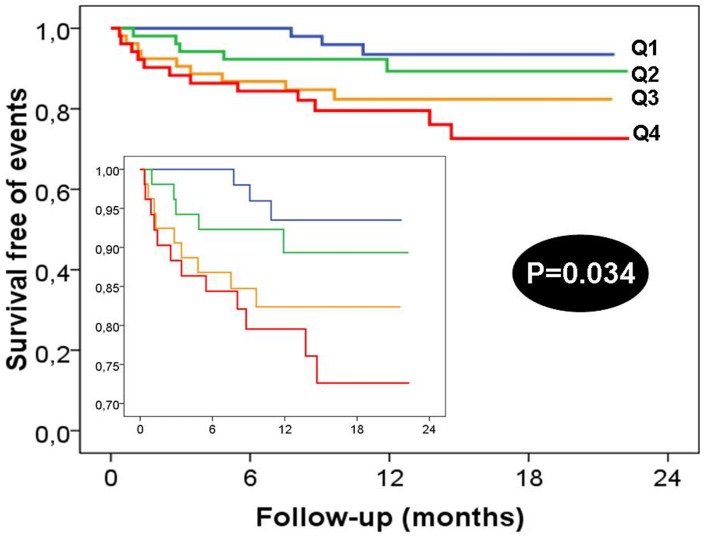
Survival Kaplan Meier curves according fluorescent AGE quartiles. Inset represent curves magnifications about survival free of events.

Regarding fluorescent AGE, the opposite was seen to occur [there was a significant increase in AGEs levels in patients with follow-up cardiac events (Figure 4), but not in patients with in-hospital events (*p* = 0.965)]. Fluorescent AGEs were not correlated with risk scores, including GRACE scores for 6-months events (r = 0.042; *p* = 0.545).

**Figure 5:**
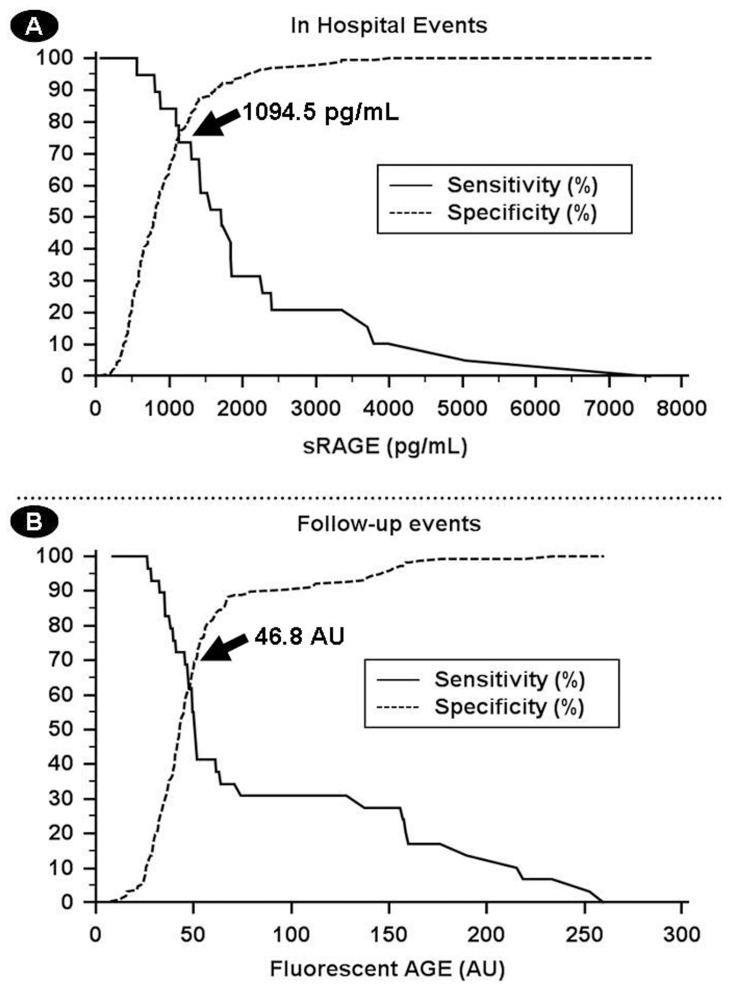
Curves of sensitivity and specificity for fluorescent AGEs and sRAGE to predict events. A) representation of sRAGE curves to predict in-hospital events and B) fluorescent AGE curves to predict follow-up events.

To predict in-hospital events, we found that the area under the curve (AUC) of sRAGE was 0.84 (95% CI: 0.75–0.93). The best cut-off level for sRAGE was 1094.5 pg/mL, with a sensitivity of 84.2% and a specificity of 73.5% (Figure 5A), as well as a negative predictive value (NPV) of 99.0% and a positive predictive value (PPV) of 15.7%. For fluorescent AGEs, the AUC was not good 0.52 (95% CI 0.41–0.64).

In relation to follow-up events, the AUC of fluorescent AGE was 0.68 (95% CI 0.57–0.79), higher than the AUC of sRAGE that was 0.55 (0.43–0.67). The cut-off level of fluorescent AGEs, which best predicts an unfavourable follow-up, was 46.8 AU, with a sensitivity of 69.0% and a specificity of 60.8% (Figure 5B), along with an NPV of 92.4% and a PPV of 28.6%.

After multivariate analysis correcting for sex, LVEF, glucose levels, haemoglobin, GRACE and SYNTAX scores, sRAGE was significantly associated with in-hospital prognosis (events risk increase 10% per 10 pg/mL), whereas fluorescent AGEs were significantly associated with long-term prognosis (events risk increase 12% per 1 AU; Table 5).

**Table 5:** Multiple regression analysis for in-hospital and follow-up events.

	In-hospital events			Follow-up events		
	OddsRatio	CI 95%	*p*	HazardRatio	CI 95%	*p*
**Female sex**	2.99	0.84–10.75	0.092	0.50	0.17–1.48	0.213
**Haemoglobin**	0.81	0.51–1.28	0.372	0.87	0.66–1.14	0.326
**Peak Troponin I**	1.00	0.99–1.01	0.712	1.01	1.00–1.01	**0.010**
**Glucose**	1.008	1.01–1.02	**0.009**	1.00	0.99–1.01	0.156
**Fluorescent AGE**	0.99	0.98–1.01	0.452	1.12	1.06–1.18	**0.001**
**sRAGE**	1.10	1.04–1.17	**0.002**	1.00	0.97–1.03	0.930
**LVEF <45%**	1.25	0.65–2.41	0.509	1.38	0.86–2.23	0.181
**High risk GRACE**	4.98	1.25–19.8	**0.023**	3.66	1.60–8.40	**0.002**
***SYNTAX score***	1.04	0.94–1.14	0.452	0.99	0.93–1.05	0.710

**LVEF**: Left Ventricular Ejection Fraction.

## Discussion

This is the first study to investigate the prognostic value of fluorescent AGEs and sRAGE in ACS patients. Elevated values of sRAGE were independently associated with a worse in-hospital prognosis, whereas high fluorescent AGE levels were associated with more events during follow-up. Both parameters reflect different risk measures, and their association with clinical outcomes is probably due to different mechanisms. Although it has been recently shown that sRAGE correlated with troponin levels in ACS [18], its prognostic value in these patients has not been studied yet.

In the context of an ACS, sRAGE is mainly a marker of inflammatory damage [8], [19]. However, to understand the role of sRAGE we must first note that sRAGE is composed of two different types of molecules, which probably have different prognostic and pathophysiological implications that may explain the controversy about the utility of sRAGE [20]. It is known that levels of sRAGE depend on the sRAGE directly secreted by cells (esRAGE) and on the sRAGE resulting from cellular transmembrane receptor cleavage (cRAGE) [1]. Controverting results have been reported about the physiopathological role of sRAGE on coronary artery disease (CAD). Falcone et al., in a study with 328 non-diabetic patients, reported an association between low levels of sRAGE and the presence of CAD [3]. On the contrary, in diabetic patients, high levels of sRAGE correlate with the presence of CAD, as reported by Nakamura et al. [21], and recently confirmed by the results of the CARDS trial [9]. This controversy could come from the absence of data about the specific contribution of each sRAGE form to the total amount of sRAGE in diabetes and CAD. Our hypothesis is that diabetes could modify the percentages of esRAGE and cRAGE and that the former could be protective whereas the latter could serve as a disease biomarker. Further investigations are needed to clarify this question. Several studies correlated sRAGE levels with inflammatory markers, such as TNF-alpha, high mobility group box 1 (HMGB1), caspase 3 or interleukin-6 [22]. The role of inflammation in the development of CAD and in plaque destabilisation is becoming increasingly well recognised. An inflammatory response is often found at the site of plaque rupture, and several investigations have correlated its degree (based on the measurement of various inflammatory biomarkers) with post-infarction mortality [23]. sRAGE was probed as an inflammatory biomarker of plaque vulnerability in patients with myocardial infarction, which correlated with higher troponin, but its prognostic value in ACS has not yet been evaluated [24].

In our study, several factors may play a role in the demonstrated association between sRAGE levels and an adverse in-hospital prognosis. Increasing sRAGE levels were clearly associated with worse Killip class, lower haemoglobin levels, more atrial fibrillation and more systolic left ventricular dysfunction, in addition to a trend to present larger myocardial infarction (higher troponin peak level) and a higher percentage of STEMI, explaining part of the increase of in-hospital mortality. Although Basta et al. [18] recently demonstrated that sRAGE was correlated with higher troponin levels in ACS, sRAGE was a predictor of in-hospital events independently of troponin levels in our study. In fact, sRAGE levels were significantly associated with the three most widely used risk scores in ACS (TIMI, PURSUIT, and GRACE).

Contrary to sRAGE, AGE is a marker of chronic oxidative stress [25], but not for acute inflammatory damage, with different characteristics in comparison to sRAGE. Whereas sRAGE is a dynamic molecule, dependent on tissue damage [7], AGE is more stable, so that its time-dependent variation curve is much flatter than that of sRAGE, as determined by the fact that AGEs take weeks to form [1]. This could explain the absence of a relationship with the in-hospital prognosis. However, since AGE reflects the degree of chronic oxidative stress and, secondarily, atherosclerotic burden [6], [26], it is reasonable to suppose that AGE should be associated with a worse medium to long-term prognosis. Several studies have demonstrated that AGEs were a marker of oxidative damage, depending on oxidative stress for its production, and likewise encouraged the formation of reactive oxygen species (ROS) [25]. ROS are important contributors to cardiovascular disease, including the events surrounding myocardial infarction. They have been implicated in numerous cardiovascular pathologies, including endothelial dysfunction or ventricular remodelling [27]. All together could serve to explain the worse prognosis of high levels of fluorescent AGEs after ACS over time. However, despite being widely tested as a marker of oxidative stress, no study analysed the prognostic and physiopathological implications of AGEs in ACS.

In our study, the association of fluorescent AGE with the presence of cardiac events was noticed during the follow-up. Patients with elevated levels of fluorescent AGEs are more prone to hypertension and smoking, which translates to a greater degree of atherosclerosis, implying a higher percentage of multi-vessel coronary disease and a higher percentage of NSTE-ACS. Although this does not indicate a worse in-hospital prognosis, it results in a greater number of events during follow-up.

### Limitations

Despite the impact and enthusiasm that our results can generate, we are aware of the limitations of our study that should be taken into account when the results are interpreted. Mainly we must consider that our study population, which included all patients admitted for ACS in the coronary care unit over a 15 month period, underwent some very strict inclusion/exclusion criteria, that supposed a significant reduction in sample size (*n* = 215) with a very selective population, which determines the statistical power of the analysis. On the other hand, fluorescent AGEs were measured globally without specifying the type of analysed AGE, whereas non-fluorescent AGEs were not analysed. Fluorescent AGEs neither was correlated with levels of markers of oxidative stress. As for the measurement of sRAGE, neither esRAGE nor cRAGE were distinguished, and neither was correlated with other markers of inflammatory damage. Despite all of this, we believe that our research is of great interest for being the first to analyse the prognostic implications of fluorescent AGE and sRAGE in the ACS spectrum.

## Conclusions

This is the first study that analyses the prognostic value of the AGE-RAGE axis in all the spectrum of ACS. As the main result, we can conclude that elevated values of sRAGE are associated with a worse in-hospital prognosis, whereas high fluorescent AGEs levels are associated with more follow-up events.

## References

[1] Brownlee M (2000) Negative consequences of glycation. Metabolism 49: 9–13. 10693913 10.1016/s0026-0495(00)80078-5

[2] Schmidt AM, Yan SD, Yan SF, Stern DM (2001) The multiligand receptor RAGE as a progression factor amplifying immune and inflammatory responses. J Clin Invest 108: 949–955. 11581294 10.1172/JCI14002 PMC200958

[3] Falcone C, Emanuele E, D’Angelo A, Buzzi MP, Belvito C, et al (2005) Plasma levels of soluble receptor for advanced glycation end products and coronary artery disease in nondiabetic men. Arterioscler Thromb Vasc Biol 25: 1032–1037. 15731496 10.1161/01.ATV.0000160342.20342.00

[4] Kislinger T, Tanji N, Wendt T, Qu W, Lu Y, et al (2001) Receptor for advanced glycation end products mediates inflammation and enhanced expression of tissue factor in vasculature of diabetic apolipoprotein E-null mice. Arterioscler Thromb Vasc Biol 21: 905–910. 11397695 10.1161/01.atv.21.6.905

[5] Park L, Raman KG, Lee KJ, Lu Y, Ferran LJ Jr, et al (1998) Suppression of accelerated diabetic atherosclerosis by the soluble receptor for advanced glycation endproducts. Nat Med 4: 1025–1031. 9734395 10.1038/2012

[6] Bucciarelli LG, Wendt T, Qu W, Lu Y, Lalla E, et al (2002) RAGE blockade stabilizes established atherosclerosis in diabetic apolipoprotein E-null mice. Circulation 106: 2827–2835. 12451010 10.1161/01.cir.0000039325.03698.36

[7] Hudson BI, Carter AM, Harja E, Kalea AZ, Arriero M, et al (2008) Identification, classification, and expression of RAGE gene splice variants. FASEB J 22: 1572–1580. 18089847 10.1096/fj.07-9909com

[8] Raucci A, Cugusi S, Antonelli A, Barabino SM, Monti L, et al (2008) A soluble form of the receptor for advanced glycation endproducts (RAGE) is produced by proteolytic cleavage of the membrane-bound form by the sheddase a disintegrin and metalloprotease 10 (ADAM10). FASEB J 22: 3716–3727. 18603587 10.1096/fj.08-109033

[9] Colhoun HM, Betteridge DJ, Durrington P, Hitman G, Neil A, et al (2011) Total soluble and endogenous secretory receptor for advanced glycation end products as predictive biomarkers of coronary heart disease risk in patients with type 2 diabetes: an analysis from the CARDS trial. Diabetes 60: 2379–2385. 21771973 10.2337/db11-0291 PMC3161327

[10] Raposeiras-Roubin S, Rodino-Janeiro BK, Grigorian-Shamagian L, Moure-Gonzalez M, Seoane-Blanco A, et al (2010) Soluble receptor of advanced glycation end products levels are related to ischaemic aetiology and extent of coronary disease in chronic heart failure patients, independent of advanced glycation end products levels: New Roles for Soluble RAGE. Eur J Heart Fail 12: 1092–1100. 20685687 10.1093/eurjhf/hfq117

[11] Dickstein K, Cohen-Solal A, Filippatos G, McMurray JJ, Ponikowski P, et al (2008) ESC Guidelines for the diagnosis and treatment of acute and chronic heart failure 2008: the Task Force for the Diagnosis and Treatment of Acute and Chronic Heart Failure 2008 of the European Society of Cardiology. Developed in collaboration with the Heart Failure Association of the ESC (HFA) and endorsed by the European Society of Intensive Care Medicine (ESICM). Eur Heart J 29: 2388–2442. 18799522 10.1093/eurheartj/ehn309

[12] Granger CB, Goldberg RJ, Dabbous O, Pieper KS, Eagle KA, et al (2003) Predictors of hospital mortality in the global registry of acute coronary events. Arch Intern Med 163: 2345–2353. 14581255 10.1001/archinte.163.19.2345

[13] Antman EM, Cohen M, Bernink PJ, McCabe CH, Horacek T, et al (2000) The TIMI risk score for unstable angina/non-ST elevation MI: A method for prognostication and therapeutic decision making. JAMA 284: 835–842. 10938172 10.1001/jama.284.7.835

[14] Morrow DA, Antman EM, Charlesworth A, Cairns R, Murphy SA, et al (2000) TIMI risk score for ST-elevation myocardial infarction: A convenient, bedside, clinical score for risk assessment at presentation: An intravenous nPA for treatment of infarcting myocardium early II trial substudy. Circulation 102: 2031–2037. 11044416 10.1161/01.cir.102.17.2031

[15] Boersma E, Pieper KS, Steyerberg EW, Wilcox RG, Chang WC, et al (2000) Predictors of outcome in patients with acute coronary syndromes without persistent ST-segment elevation. Results from an international trial of 9461 patients. The PURSUIT Investigators. Circulation 101: 2557–2567. 10840005 10.1161/01.cir.101.22.2557

[16] Sianos G, Morel MA, Kappetein AP, Morice MC, Colombo A, et al (2005) The SYNTAX Score: an angiographic tool grading the complexity of coronary artery disease. EuroIntervention 1: 219–227. 19758907

[17] Munch G, Keis R, Wessels A, Riederer P, Bahner U, et al (1997) Determination of advanced glycation end products in serum by fluorescence spectroscopy and competitive ELISA. Eur J Clin Chem Clin Biochem 35: 669–677. 9352229 10.1515/cclm.1997.35.9.669

[18] Basta G, Del Turco S, Marchi F, Navarra T, Battaglia D, et al (2011) Elevated soluble receptor for advanced glycation end product levels in patients with acute coronary syndrome and positive cardiac troponin I. Coron Artery Dis. 22: 590–594. 10.1097/MCA.0b013e32834c751f 22072229

[19] Jabaudon M, Futier E, Roszyk L, Chalus E, Guerin R, et al. Soluble form of the receptor for advanced glycation end products is a marker of acute lung injury but not of severe sepsis in critically ill patients. Crit Care Med 39: 480–488. 21220996 10.1097/CCM.0b013e318206b3ca

[20] Geroldi D, Falcone C, Emanuele E (2006) Soluble receptor for advanced glycation end products: from disease marker to potential therapeutic target. Curr Med Chem 13: 1971–1978. 16842191 10.2174/092986706777585013

[21] Nakamura K, Yamagishi S, Adachi H, Kurita-Nakamura Y, Matsui T, et al (2007) Elevation of soluble form of receptor for advanced glycation end products (sRAGE) in diabetic subjects with coronary artery disease. Diabetes Metab Res Rev 23: 368–371. 17024691 10.1002/dmrr.690

[22] Nakamura K, Yamagishi S, Adachi H, Kurita-Nakamura Y, Matsui T, et al (2007) Serum levels of sRAGE, the soluble form of receptor for advanced glycation end products, are associated with inflammatory markers in patients with type 2 diabetes. Mol Med 13: 185–189. 17592553 10.2119/2006-00090.Nakamura PMC1892766

[23] Gronholdt ML, Dalager-Pedersen S, Falk E (1998) Coronary atherosclerosis: determinants of plaque rupture. Eur Heart J 19 Suppl CC24–29. 9597422

[24] Park HJ, Baek JY, Shin WS, Kim DB, Jang SW, et al (2011) Soluble receptor of advanced glycated endproducts is associated with plaque vulnerability in patients with acute myocardial infarction. Circ J 75: 1685–1690. 21576827 10.1253/circj.cj-10-1248

[25] Bansal S, Siddarth M, Chawla D, Banerjee BD, Madhu SV, et al.. (2011) Advanced glycation end products enhance reactive oxygen and nitrogen species generation in neutrophils in vitro. Mol Cell Biochem. 10.1007/s11010-011-1114-9 22048812

[26] Selejan SR, Poss J, Hewera L, Kazakov A, Bohm M, et al (2012) Role of receptor for advanced glycation end products in cardiogenic shock. Crit Care Med 40: 1513–1522. 22430238 10.1097/CCM.0b013e318241e536

[27] Ferrari R, Agnoletti L, Comini L, Gaia G, Bachetti T, et al.. (1998) Oxidative stress during myocardial ischaemia and heart failure. Eur Heart J 19 Suppl B: B2–11. 9519346

